# MiR‐182‐5p enhances in vitro neutrophil infiltration in Kawasaki disease

**DOI:** 10.1002/mgg3.990

**Published:** 2019-10-11

**Authors:** Sung‐Chou Li, Lien‐Hung Huang, Kuang‐Jen Chien, Chao‐Yu Pan, Pei‐Hsien Lin, Yuyu Lin, Ken‐Pen Weng, Kuo‐Wang Tsai

**Affiliations:** ^1^ Genomics and Proteomics Core Laboratory Kaohsiung Chang Gung Memorial Hospital Chang Gung University College of Medicine Kaohsiung Taiwan; ^2^ Department of Pediatrics Kaohsiung Veterans General Hospital Kaohsiung Taiwan; ^3^ Institute of Biomedical Science Academia Sinica and Institute of Biomedical Informatics National Yang‐Ming University Taipei Taiwan; ^4^ Department of Medicine National Yang‐Ming University Taipei Taiwan; ^5^ Shu‐Zen Junior College of Medicine and Management Kaohsiung Taiwan; ^6^ Department of Medical Education and Research Kaohsiung Veterans General Hospital Kaohsiung Taiwan

**Keywords:** coronary artery lesion, Kawasaki disease, leukocyte transendothelial migration, miR‐182‐5p, neutrophil infiltration

## Abstract

**Background:**

Kawasaki disease (KD) patients could develop coronary artery lesion (CAL) which threatens children's life. A previous study identified KD biomarker miRNAs that could discriminate KD patients from febrile non‐KD patients. We wonder whether these KD prediction biomarkers could be further applied to predict CAL formation in KD patients.

**Methods:**

To examine this hypothesis, we conducted a meta‐analysis, miRNA mimic transfection, in vitro cell model and microarray assays.

**Results:**

We first showed that miR‐182‐5p and miR‐183‐5p kept higher levels in the KD patients with CAL than those without CAL (*p* < .05). Further machine learning alignment confirmed that CAL formation could be predicted, with an auROC value of 0.86. We further treated neutrophil cells with miR‐182‐5p mimic, followed by in vitro transendotherial migration assay. As a result, miR‐182‐5p overexpression significantly (*p* < .05) enhanced neutrophil cells to infiltrate the endothelial layer composed of human coronary artery endothelium cells. Further microarray assay and pathway enrichment analysis showed that the genes activated with miR‐182‐5p overexpression were significantly enriched in the *leukocyte transendothelial migration* pathway (kegg_pathway_194, *p* < .05).

**Conclusion:**

Therefore, our study suggested that miR‐182‐5p enhanced in vitro leukocyte infiltration by activating the leukocyte transendothelial migration pathway in CAL formation in KD.

## Graphical abstract

We showed that miR‐182‐5p could be a biomarker for CAL formation in KD. Its pathogenesis mechanism in CAL formation was also investigated.

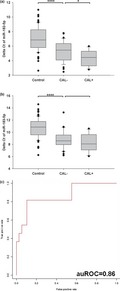

## 1 INTRODUCTION

Kawasaki disease (KD) is an infection‐like disease attacking children younger than five years old. Since first reported in 1960s, the cause of Kawasaki disease remains unknown (Burns & Glodé, 2004). The manifestation of Kawasaki disease includes fever for at least 5 days, which is the earliest and most obvious syndrome. Almost 20% of KD patients may develop severe coronary artery lesion (CAL) without early diagnosis and timely treatment (Burns & Glodé, 2004). Therefore, KD is the leading cause of acquired heart disease in young children in developed countries (Kuo, Yang, Chang, Ger, & Hsieh, 2012). The detection of KD within the first 10 days of fever onset followed by high dose intravenous immunoglobulin (IVIG) can greatly reduce the risk of severe CAL (Burns & Glodé, 2004).

So far, the detection of KD currently relies on clinical physicians’ judgment based on the AHA 2004 (Newburger et al., 2004) or JCS 2008 diagnostic criteria. Infectious diseases caused by other pathogens, such as staphylococcal or streptococcal, may also cause symptoms similar to the ones of KD (Burns & Glodé, 2004), which makes it difficult to accurately diagnose KD. Therefore, several studies worked on developing biomarkers and/or novel detection methodology of KD. Burns et al identified clinical and epidemiologic characteristics of KD (Burns et al., 1991). Wright and colleagues developed a minimal whole‐blood gene expression signature which effectively diagnosed KD (Wright et al., 2018). These studies facilitated the accurate and early detection of KD, largely reducing the incidence rate of CAL formation by KD.

However, even with early diagnosis and timely administration of IVIG, a fraction of KD patients still develop CAL, which may cause death of patients. Such a phenomenon highlighted the necessity of early prediction of CAL formation in KD patients. In our previous study, we identified 10 miRNAs which were differentially expressed between fever control subjects and KD patients (Kuo et al., 2016). We were interested in finding whether these KD biomarker miRNAs were able to predict CAL formation. In other words, they were not only KD diagnosis biomarkers but also KD prognosis ones. Among the 10 KD biomarker miRNAs, we were especially interested in miR‐182‐5p; it was first reported to play important roles in urinary cancers. miR‐182‐5p was shown to promote cell invasion and proliferation in prostate cancer (Hirata et al., 2013), so it served as a useful biomarker for high grade prostate cancer (Tsuchiyama et al., 2013). In bladder cancer, miR‐182‐5p also functioned as an oncogene increasing the proliferation, migration and invasion of cancer cells (Hirata et al., 2012). In addition, a similar result was also observed in acute myeloid leukemia (Zhang, Zhang, Shi, & Yin, 2018). These studies highlighted miR‐182‐5p's power to promote cell migration. Therefore, we hypothesized that miR‐182‐5p could also promote leukocyte transendothelial migration, triggering CAL formation in KD.

To examine this hypothesis, we enrolled KD patients with CAL complication and examined miR‐182‐5p and miR‐183‐5p using qPCR. We first confirmed that miR‐182‐5p and miR‐183‐5p were differentially expressed in KD patients without CAL and KD patients with CAL. Further analysis with machine learning algorithm demonstrated a high performance of predicting CAL formation using the expression data of the two miRNAs. Finally, we used an in vitro cell model to demonstrate that miR‐182‐5p and miR‐183‐5p enhanced leukocyte infiltration and the underlying mechanism was also investigated.

## 2 MATERIALS AND METHODS

### 2.1 Clinical sample information and ethical compliance

This study was approved by the institutional ethics board (IRB number: 201600330B0C601) of Kaohsiung Chang Gung Memorial Hospital. All subjects or their guardians signed the informed consent forms. In this study, we enrolled 11 KD patients in the acute phase. These patients had an average age of 1.66% and 63.6% of the subjects were male. As a result, there was no significant difference in age or gender between the newly enrolled subjects and the subjects in the previous study (H. C. Kuo et al., 2016). All of the enrolled KD patients followed the American Heart Association (AHA) 2017 diagnostic criteria (McCrindle et al., 2017) and were detected as CAL with echocardiography examination. To exclude the influences of drugs, all subjects were subject to blood collection before IVIG, aspirin or antibiotics administration.

### 2.2 Cell culture

HL‐60 cells (BCRC No. 60,027) were maintained in Iscove's modified Dulbecco's medium supplemented with 20% fetal bovine serum, 4 mM L‐glutamine, and 1.5 g/L sodium bicarbonate and incubated at 37°C in 5% CO_2_. Then, as suggested in a previous study (Walsh, 2009), HL‐60 cells were differentiated into neutrophil‐like cells by stimulation with 1.3% DMSO (Sigma‐Aldrich). Human coronary endothelial cells (HCAEC, CC‐2585, Clonetics, Lonza) were maintained in EBM‐2 medium (CC‐3156, Clonetics, Lonza) supplemented with EGM‐2 MV SingleQuots (CC‐4147, Clonetics, Lonza) and incubated at 37°C in 5% CO_2_.

### 2.3 Overexpression of miRNA mimic in neutrophil cells

miRNA mimic Syn‐hsa‐miR182‐5p (MSY0000259) and Syn‐hsa‐miR183‐5p (MSY0000261) were obtained from QIAGEN. For miRNA mimic transfection, 2*10^6^ neutrophil cells were seeded in a 6‐well plate and each well contained 200 µl complete medium. After 24 hr, 6 µl miRNA mimic (20 µM) and 12 µl HiPerFect transfection reagent (QIAGENT) were mix in 200 µl culture medium without serum added. After 6 hr, 800 µl culture medium was added into 6‐well plate and the cells were cultured. Finally, 24 hr after transfection, the cells were harvested for further analysis.

### 2.4 Transendothelial migration assay

In this study, we used an upper chamber and an lower chamber to comprise the kit for leukocyte transendothelial migration assay. The bottom membrane of the upper chamber was coated with human coronary artery endothelium cells (HCAECs) to form an endothelium layer. During the assay, the leukocyte cells migrate through the endothelial layer from the upper chamber to the lower chamber. Therefore, the upper chamber, endothelium layer and lower chamber simulated the lumen, endothelium layer and intima of vessel, respectively.

For the transendothelial migration assay, 2 × 10^5^ HCAECs were seeded in gelatin‐coated 24‐well hanging inserts (also called upper chamber, 3 μm, PET, Merck). After 24 hr, the inserts were put into 24‐well culture plates. 1 × 10^5^ miRNA‐transfected neutrophil cells were placed in the inserts which were further moved into the 24‐well culture plates containing 600 µl medium with 200 nM fMLP (Sigma‐Aldrich). Two hours after migration, the cells were collected from the 24‐well culture plates and labeled with CD15‐FITC (340,703, BD), followed by analysis with a LSRⅡflow cytometer (BD Biosciences).

### 2.5 RNA extraction and Real‐Time qPCR assay

For gene quantification, total RNA was extracted using a mirVanaTM miRNA Isolation Kit (Ambion). Following total RNA extraction, 0.1 µg total RNAs were reverse transcribed to cDNA using a High‐Capacity cDNA Reverse Transcription Kit (Applied Biosystems). We performed Real‐Time qPCR using Fast SYBR^®^ Green Master Mix system and conducted with 7,500 Fast Real‐Time PCR System (Applied Biosystems, CA, USA). The sequences of the primers used are as follows:

18S: forward primer (5ʹ‐ GTAACCCGTTGAACCCCATT ‐3ʹ) and reverse primer (5ʹ ‐ CCATCCAATCGGTAGTAGCG ‐3ʹ); ITGB1: forward primer (5ʹ ‐ GTGAATGTGAATGCCAAAGC‐3ʹ) and reverse primer (5ʹ ‐ ATCCATGTCTTCACTGTTAACT‐3ʹ); CXCR4: forward primer (5ʹ ‐ TTGTCATCCTGTCCTGCTATT‐3ʹ) and reverse primer (5ʹ ‐ ACACCCTTGCTTGATGATT‐3ʹ). ITGAM: forward primer (5ʹ ‐ TTAACATCACCAACGGAGC‐3ʹ) and reverse primer (5ʹ ‐ GGATTTCTCACTGCGGAAG‐3ʹ).

For RNU6B (U6) and miRNA quantification, the RNAs were reverse transcribed to cDNA using a TaqMan MicroRNA Reverse Transcription Kit (Applied Biosystems) and TaqMan^®^ MicroRNA Assays. The AB Assay IDs of RNU6B, miR‐182‐5P and miR‐183‐5P were 001093, 002334 and 002269, respectively. The expression was quantified using TaqMan^®^ Fast Universal PCR Master Mix (2×), No AmpErase^®^ UNG (Applied Biosystems) and 7,500 Fast Real‐Time PCR System (Applied Biosystems).

The ΔCt values of miR‐182‐5p and miR‐183‐5p in Control set (fever control subjects, *n* = 70) and CAL‐ KD set (KD subjects without CAL, *n* = 50) were downloaded from a previous study (Kuo et al., 2016). The ΔCt values in CAL + KD set (KD subjects with CAL, *n* = 11) were determined by conducting qPCR assays on the subjects enrolled for this study. We used U6 as the internal control to calibrate the expression levels of miR‐182‐5p and miR‐183–5‐p.

## 3 RESULTS

### 3.1 miR‐182‐5p and miR‐183‐5p could be potential biomarkers to predict CAL formation in KD

**Figure 1:**
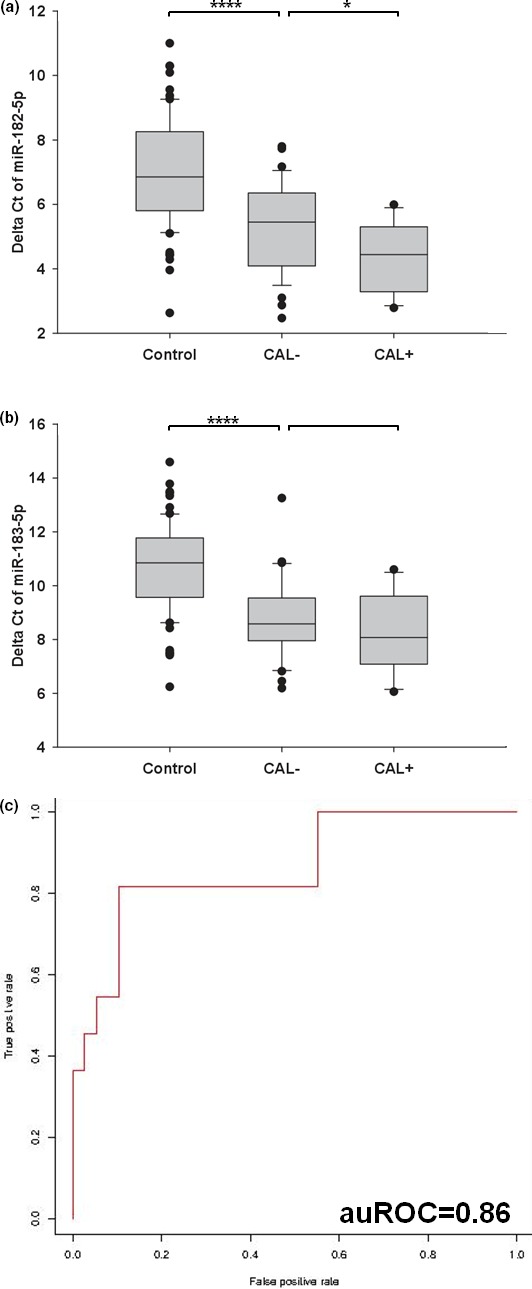
The expression profiles and CAL prediction potential of miR‐182‐5p and miR‐183‐5p. (a,b) We used a boxplot to illustrate the expression profiles of miR‐182‐5p and miR‐183‐5p in Control, CAL‐ KD and CAL + KD sets. All ΔCt values were derived by using U6 as the internal control. (c) We used SVM to derive a discrimination model. With fivefold cross validation specified, we first derived the best parameters, gamma = 1 and cost = 2. With gamma and cost specified, we derived the discrimination model with an auROC value = 0.86. **** and * denoted *p* < .0001 and *p* < .05, respectively

In our previous study, we identified several miRNAs that could distinguish fever control subjects (FC, the subjects with fever but not diagnosed as KD) from KD patients (Kuo et al., 2016). These miRNAs could serve as KD biomarkers. Since coronary artery lesion (CAL) is a severe complication of KD, we were interested in finding whether these KD biomarker miRNAs could further predict CAL formation, functioning as CAL biomarkers. Therefore, we further compared the expression levels of these biomarker miRNAs between the KD samples without CAL complication (CAL‐ KD), and the KD samples with CAL complication (CAL + KD). As shown in Figure 1a, miR‐182‐5p was not only differentially expressed between FC and CAL‐ KD samples but also differentially expressed between CAL‐ KD and CAL + KD samples. For miR‐183‐5p, although not yet significant, CAL + KD patients tended to keep higher level than CAL‐ KD ones (Figure 1b). Such result implied that miR‐182‐5p and miR‐183‐5p not only were the biomarkers of KD onset but also could be the ones of CAL formation.

To examine whether the two miRNAs can distinguish CAL‐ KD from CAL + KD samples, we used support vector machine (SVM) to derive a prediction model. SVM is one type of machine learning algorithm and is good in dealing with binary classification problems (Li et al., 2010; Xue et al., 2005). So, SVM meets the demands of many biomedical problems, e.g. treatment versus control or disease versus normal (Kain et al., 2013; Kvon et al., 2014). In this study, we applied the libsvm (Chang & Lin, 2001) module for this job. Therefore, by using the delta Ct values of the two miRNAs as the training data of SVM, we derived a prediction model (Figure 1c), the auROC value, 95% confidence interval and p‐value which were 0.86, (0.72, 1.00) and 0.0001, respectively, by referring to a previous study (Hanley & McNeil, 1982). Such a result implied the applicability of using miR‐182‐5p and miR‐183‐5p to predict CAL formation in KD patients.

Developing novel disease diagnosis and prognosis methods is the point of precision medicine, enhancing medical outcome and reducing medical waste. Although the auROC value of the CAL prediction model was high, the sample size of the training set was pretty low, making the result not robust enough. Therefore, this prediction model is an explorative study only. We need to enroll more subjects and examine more samples for deriving a more robust and unbiased prediction model.

### 3.2 *MiR‐182‐5p and miR‐183‐5p enhanced *in vitro* neutrophil cells infiltration*

Next, we were interested in the correlation between miRNAs' function and CAL formation. CAL is a type of vascular inflammation and results from leukocyte infiltration into coronary artery. Previous studies concluded that T lymphocyte, macrophage and neutrophil cells contributed to the infiltration in KD (Brown et al., 2001; Takahashi, Oharaseki, Naoe, Wakayama, & Yokouchi, 2005). In addition, KD does not have a good in vitro cell model. Therefore, we mimicked the in vitro leukocyte transendothelial migration assay proposed in a previous study (Walsh, 2009) with a few modifications. As demonstrated in the Materials and Method section, we coated the bottom membrane of the upper chamber with human coronary artery endothelium cells (HCAECs) to form an endothelium layer. As a result, the upper chamber, endothelium layer and lower chamber simulated the lumen, endothelium layer and intima of vessel, respectively. This methodology was also applied in our previous study to examine the pathogenesis roles of S100A family genes in KD (Huang et al., 2018).

**Figure 2:**
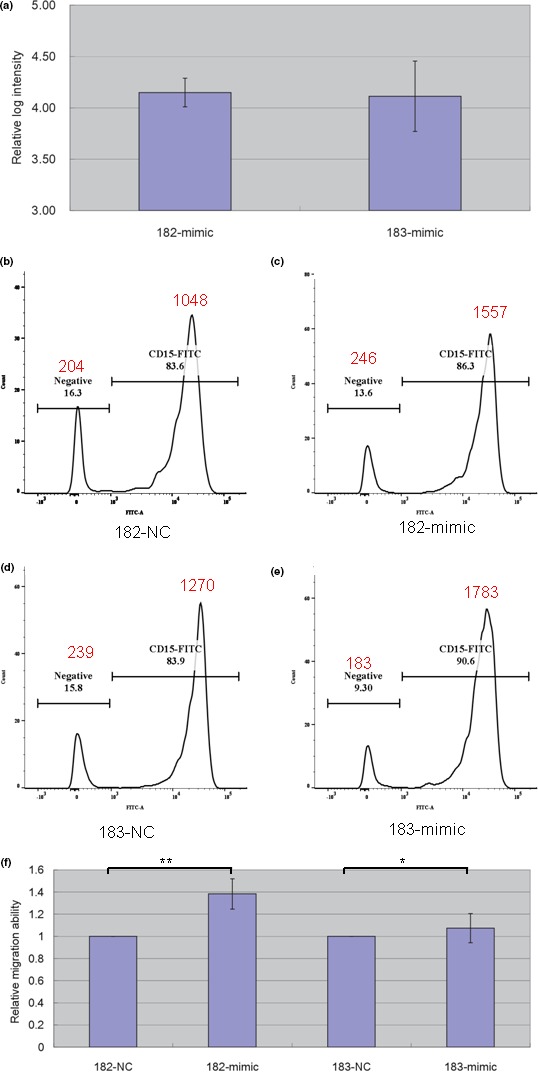
The results of miRNA mimic transfection and transendothelial migration assay. (a) We transfected neutrophil cells with scrambled control (182‐NC and 183‐NC), miR‐182‐5p mimic (182‐mimic) or miR‐183‐5p mimic (183‐mimic). (b,c,d,e) Examples of flow cytometry quantification on the infiltrating neutrophil cells. CD15‐FITC peaks denoted the numbers of infiltrating neutrophil cells. (F) Compared with scrambled control (182‐NC), transfecting miR‐182p‐5p mimic allowed more neutrophil cells to penetrate the endothelium layer and to move down to the lower chamber, enhancing in vitro neutrophil infiltration. A similar effect was also observed for miR‐183‐5p. The data were derived from three independent assays and demonstrated as mean ± *SD*. ** denoted *p* < .01

To examine the effects of miR‐182‐5p and miR‐183‐5p on CAL formation, we first transfected neutrophil cells with miR‐182p‐5p mimic, miR‐183‐5p mimic or scrambled control, followed by qPCR assay. As shown in Figure 2a, in comparison to scrambled control, transfecting miR‐182p‐5p mimic and miR‐183‐5p mimic both succeeded in enhancing their expression intensities by more than ten thousand times. Then, we subjected the transfected neutrophil cells to in vitro transendothelial migration assays. Figure 2b,c illustrated one run of 183‐mimic versus scrambled control comparison where more neutrophil cells infiltrated the HCAEC layer with miR‐182‐5p overexpression. Similar result was observed with miR‐183‐5p overexpression (Figure 2d,e). Figure 2f showed that increasing miR‐182‐5p and miR‐183‐5p both significantly enhanced neutrophil infiltration (*p* < .01 for miR‐182‐5p and *p* < .05 for miR‐183‐5p).

### 3.3 The gene altered by miR‐182‐5p was involved in immune‐related responses

**Figure 3:**
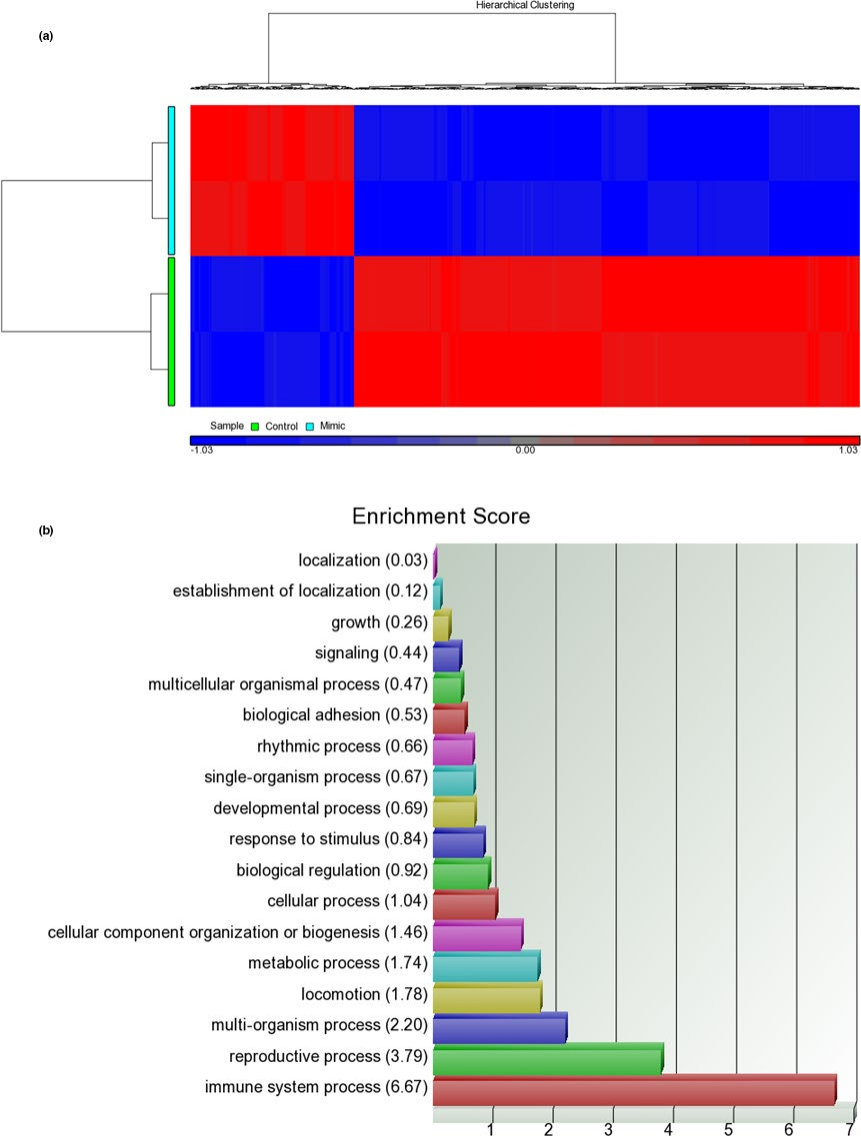
The results of microarray assays. We collected RNA samples from the neutrophil cells transfected with miR‐182‐5p mimic or scramble control, followed by microarray assay with Affymetrix Clariom D chips. (a) Based on the criteria *p* < .01, there were 644 and 183 genes down‐regulated and up‐regulated, respectively. (b) All significant genes were subject to GO assay. The result showed that immune‐related process had the highest score, which was consistent with the content presented in Table 1 and the nature of KD

Next, we were interested in the possible pathways through which miR‐182‐5p promoted transendothelial migration in neutrophil cells. To find this, we conducted microarray assays on the cells transfected with miR‐182p‐5p mimic or scrambled control. Figure 3a shows that most of the significantly altered genes were down‐regulated (644 down‐regulated genes versus 183 up‐regulated genes) with miR‐182‐5p overexpression, which could reflect the down‐regulation nature of miRNA. Figure 3a also shows that the samples transfected with scrambled control and miR‐182‐5p mimic were separated well based on these significantly altered genes.

To understand the possible functions of these significantly altered genes, we analyzed them with gene ontology (GO) assay. As illustrated in Figure 3b, these altered genes were mainly involved in immune‐related responses. In addition, Table 1 also demonstrates that among the top ten significant GO terms, immune‐related activities dominated over others. Therefore, the genes altered by miR‐182‐5p were involved in immune‐related responses, which was consistent with the nature of KD, an acute immune response.

**Table 1:** The result of GO analysis on all altered genes.

Function	Type	Enrichment Score	Enrichment *p*‐value	GO ID
Spermatid nucleus differentiation	BP^a^	6.767	.001	7,289
Immune response‐regulating signaling pathway	BP	6.696	.001	2,764
Immune system process	BP	6.665	.001	2,376
Regulation of innate immune response	BP	6.418	.002	45,088
Immune response‐regulating cell surface receptor signaling pathway	BP	6.205	.002	2,768
Mucosal‐associated lymphoid tissue development	BP	5.909	.003	48,537
Peyer's patch development	BP	5.909	.003	48,541
Regulation of Golgi to plasma membrane protein transport	BP	5.909	.003	42,996
Regulation of establishment of cell polarity	BP	5.692	.003	2,000,114
Acrosome assembly	BP	5.692	.003	1,675

We conducted GO assay on the significantly altered genes with Partek. Only the top ten most significant GO terms are listed in this table.

a Biological process.

### 3.4 Overexpression of miR‐182‐5p activated the leukocyte trasendothelial migration pathway

**Figure 4:**
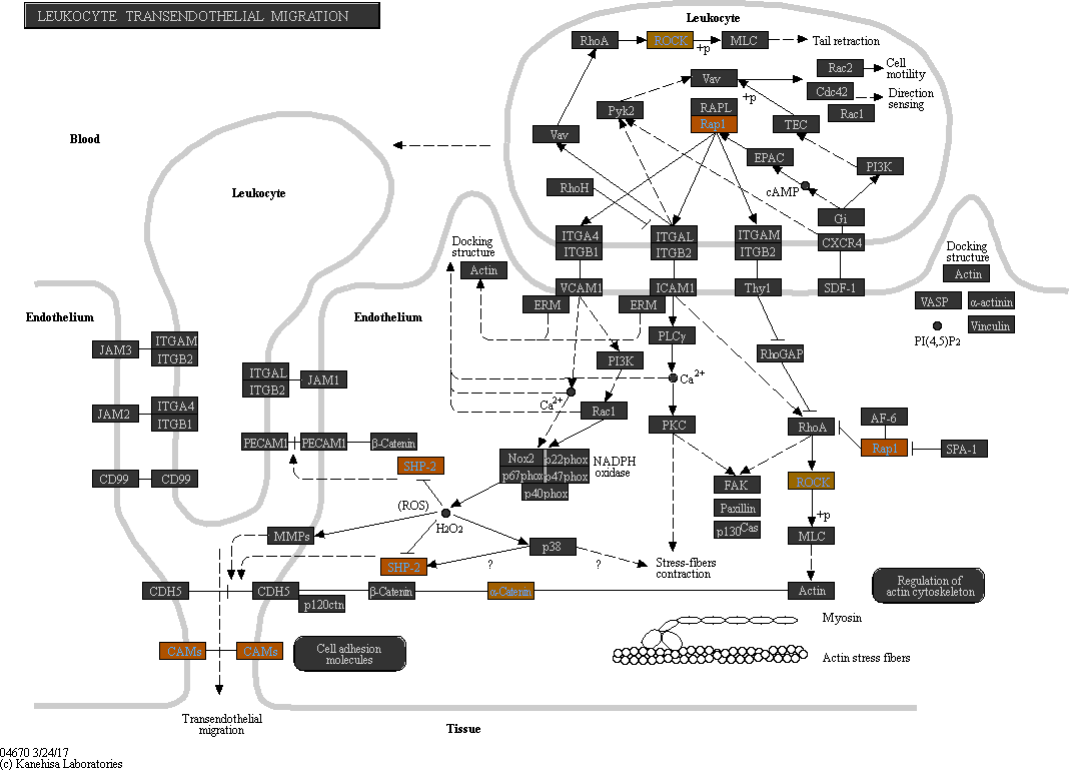
The results of pathway enrichment analysis. The significant genes were then divided into up‐regulated and down‐regulated sets. (a) We subjected the up‐regulated gene to pathway enrichment analysis with Partek. The most significant pathway identified was the *leukocyte transendothelial migration* pathway (*p* = .002) and the genes involved in this pathway are highlighted with a brown box

**Figure 5:**
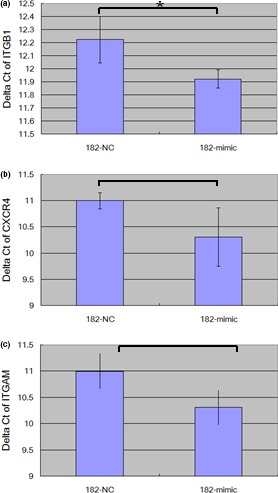
The results of qPCR validation. We used qPCR to examine the genes responsible for adhesion with endothelium cells. As a result, ITGB1, the downstream target of Rap1, was also significantly up‐regulated with miR‐182‐5p overexpression. The data were derived from three independent assays and demonstrated as mean ± *SD*

We further divided the differentially expressed genes into two sets, up‐regulated or down‐regulated, and subjected to pathway enrichment analysis. As shown in Table 2, the most significant pathway enriched by the up‐regulated genes was the *Leukocyte transendothelial migration* pathway. Since coronary artery lesion is triggered by leukocyte infiltration in coronary artery, the result of pathway enrichment analysis matched the clinical outcome of KD. Therefore, we further investigated the “*Leukocyte transendothelial migration*” pathway. As illustrated in Figure 4a, the genes in red box denote the significant genes up‐regulated with miR‐182‐5p overexpression. Within this pathway, Rap1 is the upstream positive regulator of many genes responsible for the adhesion between leukocyte and endothelial cells. Although these adhesion genes were not altered by microarray examination, we used qPCR to examine their expressions. As shown in Figure 5a, ITGB1 was significantly up‐regulated with miR‐182‐5p overexpression. Although not yet significant for CXCR4 and ITGAM, the expression tendencies were observed (Figure 5b,c). In summary, overexpression of miR‐182‐5p activated the *leukocyte trasendothelial migration* pathway.

**Table 2:** The result of pathway enrichment analysis on the up‐regulated genes with miR‐182‐5p overexpression.

Pathway name	Enrichment score	Enrichment *p*‐value	Pathway ID
Leukocyte transendothelial migration	6.404	.002	kegg_pathway_194
Hepatitis C	5.749	.003	kegg_pathway_280
RIG‐I‐like receptor signaling pathway	5.485	.004	kegg_pathway_181
Herpes simplex infection	4.521	.011	kegg_pathway_287
Protein processing in endoplasmic reticulum	3.208	.040	kegg_pathway_140
Necroptosis	3.208	.040	kegg_pathway_153
Renal cell carcinoma	3.192	.041	kegg_pathway_296
Epithelial cell signaling in Helicobacter pylori infection	3.192	.041	kegg_pathway_266
Adipocytokine signaling pathway	3.166	.042	kegg_pathway_224
NOD‐like receptor signaling pathway	3.164	.042	kegg_pathway_180
RNA transport	3.035	.048	kegg_pathway_99

We conducted pathway enrichment analysis on the significantly up‐regulated genes with Partek. As a result, 11 significant pathways were identified.

For the down‐regulated genes, we also conducted the same pathway enrichment analysis and the result is available in Table 3.

**Table 3:** The result of pathway enrichment analysis on the down‐regulated genes with miR‐182‐5p overexpression.

Pathway name	Enrichment score	Enrichment *p*‐value	Pathway ID
Toxoplasmosis	7.8055	.0004	kegg_pathway_276
Arrhythmogenic right ventricular cardiomyopathy (ARVC)	7.3421	.0006	kegg_pathway_323
Olfactory transduction	6.3449	.0018	kegg_pathway_209
Th1 and Th2 cell differentiation	6.3047	.0018	kegg_pathway_187
Th17 cell differentiation	5.8026	.0030	kegg_pathway_188
*Staphylococcus aureus* infection	5.6723	.0034	kegg_pathway_278
Leishmaniasis	4.8783	.0076	kegg_pathway_272
Pathways in cancer	4.7909	.0083	kegg_pathway_289
Pertussis	4.6960	.0091	kegg_pathway_270
Glycosphingolipid biosynthesis ‐ lacto and neolacto series	4.5626	.0104	kegg_pathway_61
Hypertrophic cardiomyopathy (HCM)	4.5264	.0108	kegg_pathway_322
Asthma	4.4291	.0119	kegg_pathway_314
Dilated cardiomyopathy (DCM)	4.3375	.0131	kegg_pathway_324
Colorectal cancer	4.3375	.0131	kegg_pathway_295
Rheumatoid arthritis	4.3074	.0135	kegg_pathway_318
Hematopoietic cell lineage	4.1070	.0165	kegg_pathway_184
Progesterone‐mediated oocyte maturation	4.1070	.0165	kegg_pathway_218
Influenza A	4.0758	.0170	kegg_pathway_283
Allograft rejection	4.0277	.0178	kegg_pathway_319
Herpes simplex infection	3.8791	.0207	kegg_pathway_287
Toll‐like receptor signaling pathway	3.8479	.0213	kegg_pathway_179
Graft‐versus‐host disease	3.8336	.0216	kegg_pathway_320
Type I diabetes mellitus	3.7442	.0237	kegg_pathway_237
MAPK signaling pathway	3.7098	.0245	kegg_pathway_115
Focal adhesion	3.6959	.0248	kegg_pathway_168
Intestinal immune network for IgA production	3.5782	.0279	kegg_pathway_195
Regulation of actin cytoskeleton	3.5551	.0286	kegg_pathway_213
Autoimmune thyroid disease	3.3913	.0337	kegg_pathway_315
Systemic lupus erythematosus	3.3077	.0366	kegg_pathway_317
Viral myocarditis	3.1918	.0411	kegg_pathway_325
Cell adhesion molecules (CAMs)	3.1074	.0447	kegg_pathway_170
PI3K‐Akt signaling pathway	3.0555	.0471	kegg_pathway_146

We conducted pathway enrichment analysis on the significantly down‐regulated genes with Partek. As a result, 32 significant pathways were identified.

## 4 DISCUSSION

Our study demonstrated the role of miR‐182‐5p in the pathogenesis of CAL formation in KD. As presented in the Introduction section, miR‐182‐5p was an oncegene in many urinary cancers. Interestingly, miR‐182‐5p functioned as a tumor repressor in renal cancer by inhibiting cell proliferation, colony formation through either diminished MALAT‐1 expression (Kulkarni et al., 2018) or activating AKT/FOXO3a signaling pathway (Xu et al., 2014). In addition, it was also found to promote hepatocellular carcinoma progression by repressing FOXO3a (Cao et al., 2018) or promoting glioma tumorigenesis by STAT3 activation (Xue et al., 2016). Recent studies also revealed that miR‐182‐5p participated in a wide range of biological activities, e.g. regulating nerve injury‐induced nociceptive hypersensitivity (Zhou et al., 2017) or inhibiting chondrogenesis (Bai, Yin, Zhao, Li, & Wu, 2018). Our study enhanced our knowledge of miR‐182‐5p's biological roles, demonstrating that leukocyte transendothelial migration was activated by miR‐182‐5p in CAL formation in KD. To the best of our knowledge, this is the first study to show the close relationship of miR‐182‐5p and CAL formation in KD.

Coronary artery lesion (CAL) is a type of vascular inflammation and results from leukocyte infiltration in coronary artery. Although it brings severe effects on the patients, even death (Rowley et al., 2014), previous KD‐related studies seldom investigated the pathogenesis of CAL triggered by KD. In 2001, Brown et al examined clinical CAL samples from patients who died of KD. Using immunohistochemical staining, they found that CD8 T lymphocytes and macrophages infiltrated into the intima layer (Yang, Wang, Li, Tiao, & Huang, 2017). In 2005, Takahashi and colleagues did similar studies and further quantified the types of infiltrating WBCs. They concluded that neutrophils also contributed to the infiltration in addition to T lymphocyte and macrophages (Yang et al., 2016). In 2012, Weng et al found that neutrophil activation has been suggested to play an important role in KD pathogenesis (Weng et al., 2012). Since neutrophil was also blamed for CAL formation in KD and neutrophil accounted for more than 50% population of all WBCs, we used neutrophil cells in this study to evaluate transendothelial migration.

In this study, we conducted a pathway enrichment assay to identify the possible pathway through which miR‐182‐5p enhanced leukocyte infiltration, implying induction of CAL formation. Although the activated pathway "leukocyte transendothelial migration" explained the function of miR‐182‐5p well, other pathways also deserved discussion. Among the pathways presented in Table 3, we were interested in the "Th1 and Th2 cell differentiation" pathway. The variations of Th1 and Th2 cytokines were usually investigated in KD‐related studies. Kimura et al. conducted qPCR to examine IFN‐gamma and IL‐4 in the PBMC from KD and healthy control. They found that these Th1/Th2 cytokine genes tend to be lower in KD patients (Kimura et al., 2004). With the prevalence of IVIG administration, KD patients with CAL were usually thought to be nonresponsive to IVIG. Wang and colleagues found that, after IVIG administration, serum Th1 and Th2 cytokines tend to be higher in the CAL + KD patients (nonresponsive to IVIG) than in the CAL‐ KD patients (Wang et al., 2013). Such conclusion seemed to be inconsistent with our finding although we examined the mRNA levels of Th1 and Th2 differentiation genes and Wang examined the serum cytokines. In conclusion, the involvement of the inactivated pathways in KD deserves more attention and investigation.

Before infiltration, leukocytes must first adhere to the endothelial cells of vessels. According to Figure 4, ITGA4, ITGAL, ITGAM, ITGB1, ITGB2 and CXCR4 are the leukocyte genes responsible for adherence with endothelial cells. The expression variations of these six genes are of interest. However, we succeeded in designing the qPCR primers only for CXCR4, ITGAM and ITGB1. As a result, we conducted qPCR assays on these three genes.

## 5 CONCLUSION

In this study, we were interested in identifying biomarkers that could predict CAL formation in KD patients. By meta‐analysis, we first identified a CAL prediction model based on miR‐182‐5p and miR‐183‐5p expression. Although such a high‐performance model was derived based on a limited sample size, it deserves further attention. In addition, by using an in vitro cell model and microarray assays, we concluded that miR‐182‐5p overexpression enhanced neutrophil infiltration by activating the *leukocyte transendothelial migration* pathway.

## CONFLICTS OF INTEREST

The authors declare no commercial or financial conflict of interest.

## Data Availability

All microarray data were submitted to NCBI GEO. Please refer to http://www.ncbi.nlm.nih.gov/geo/query/acc.cgi?acc=GSE120431 for the microarray data.
